# Referent Cueing, Position, and Animacy as Accessibility Factors in Visually Situated Sentence Production

**DOI:** 10.3389/fpsyg.2020.02111

**Published:** 2020-08-27

**Authors:** Yulia Esaulova, Martina Penke, Sarah Dolscheid

**Affiliations:** Department of Special Education and Rehabilitation, University of Cologne, Cologne, Germany

**Keywords:** accessibility, sentence production, eye tracking, cueing, animacy, structural choices, left-to-right preferences

## Abstract

Speakers’ readiness to describe event scenes using active or passive constructions has previously been attributed—among other factors—to the accessibility of referents. While most research has highlighted the accessibility of agents, the present study examines whether patients’ accessibility can be modulated by means of visual preview of the patient character (derived accessibility), as well as by manipulating the animacy status of patients (inherent accessibility). Crucially, we also examined whether effects of accessibility were amenable to the visuospatial position of the patient by presenting the patient character either to the left or to the right of the agent. German native speakers were asked to describe drawings depicting event scenes while their gaze and speech were recorded. Our results show that making patients more accessible using derived and inherent accessibility factors led to more produced passives, shorter speech onsets, and a reduction of fixations on patients. Complementing previous research on agent accessibility, our findings demonstrate that the accessibility of patients affected both sentence production and looking behavior. While effects were observed for both inherent and derived accessibility, they appeared to be more pronounced for the latter. Regarding character position, we observed a significant effect of position on participants’ gaze patterns and structural choices, suggesting that position itself can be considered an accessibility-related factor. Importantly, the position of a patient also interacted with our manipulation of its accessibility via visual preview. Participants produced more passives after preview than no preview for left-positioned but not for right-positioned patients, demonstrating that effects of patient accessibility (i.e., visual preview) were susceptible to character position. A similar interaction was observed for participants’ viewing patterns. These findings provide the first evidence that the position of a referent is a factor that interacts with other accessibility-related factors (i.e., cueing), emphasizing the need of controlling for position effects when testing referent accessibility.

## Introduction

When speakers describe an event that they observe—such as a snowball fight between a boy and a girl—they may find putting certain elements of the scene into words easier than others. This can be reflected in both the structure of the produced utterance and how quickly it is formulated. To illustrate this, imagine you observe a snowball hitting the boy. If you were to describe this event, you could produce an utterance like “The snowball is hitting the boy,” with the snowball being realized as the subject of the sentence. Alternatively, you could produce a passive construction like “The boy is being hit by the snowball.” In this case, the boy (i.e., the patient or undergoer) would take up the subject role. Speakers’ preferences to assign the subject position to one of the two referents (the boy or the snowball) depend on the referents’ accessibility. According to Bock and Warren, accessibility “is the ease with which the mental representation of some potential referent can be activated in or retrieved from memory” (Bock and Warren, 1985, p. 50). That is, referents or referring expressions can be more or less “accessible” or “active” in the speaker’s mind. For instance, there is ample evidence that animate referents tend to be more accessible than inanimate ones and therefore are preferably realized as sentential subjects (e.g., Bock et al., 1992; McDonald et al., 1993; Prat-Sala and Branigan, 2000; Van Nice and Dietrich, 2003; Tanaka et al., 2011). In the abovementioned example, speakers would thus be more inclined to produce a sentence like “The boy (+animate) was hit by the snowball (−animate).” A similar effect can be observed regarding the imageability of referents (Bock and Warren, 1985). For instance, participants tend to assign higher grammatical roles to concrete referents (e.g., table) than to abstract ones (e.g., time), demonstrating that concrete referents are more accessible (Bock and Warren, 1985). Another facet of accessibility, namely, lexical accessibility or “the ease with which the representations of word forms can be recovered from memory” (Bock and Warren, 1985, p. 52), can likewise influence speakers’ structural choices. For instance, van de Velde and colleagues examined whether referents that can be described with a small number of nouns (i.e., high codability) versus those described with a wider range of nouns (i.e., low codability) affected sentence production (van de Velde et al., 2014, also see Konopka and Meyer, 2014). They found that speakers produced fewer active sentences when the agent of an event was difficult to name (i.e., when character codability was low) than when its codability was high (van de Velde et al., 2014), providing further evidence for effects of accessibility on structural choices.

Taken together, a wealth of psycholinguistic studies has demonstrated that the accessibility of a referent can affect speakers’ structural preferences as well as the time course of sentence formulation (e.g., Bock and Warren, 1985; van de Velde et al., 2014; Ganushchak et al., 2017). While quite a number of different attributes are subsumed under the term *accessibility*, these studies have in common that they all focus on properties of a referent or referring expressions (e.g., animate vs. inanimate, abstract vs. concrete, high vs. low codability; see, e.g., Bock and Warren, 1985; Bock et al., 1992; McDonald et al., 1993; Prat-Sala and Branigan, 2000; Christianson and Ferreira, 2005; Norcliffe et al., 2015). Other studies, however, have examined the accessibility of a referent as a function of context (sometimes also termed “derived accessibility”; see Prat-Sala and Branigan, 2000). For instance, Konopka (2014) asked participants to describe pictures of simple events such as a frog catching a fly. Before viewing the pictures, participants were presented with short sentences that mentioned either the agent (the frog) or the patient (the fly). When the agent was mentioned prior to an event, speakers were faster in producing subject-verb-object (SVO) sentences, suggesting that the linguistic context raised the accessibility of the agent (Konopka, 2014). Converging evidence for this observation comes from a study by Ganushchak et al. (2017). Prior to describing pictures of transitive events, participants heard a story where the agent (e.g., the frog) was either mentioned explicitly, implicitly or not mentioned at all (Ganushchak et al., 2017). While Ganushchak and colleagues did not observe any effects of accessibility on speakers’ speech onsets, they found differences in speakers’ looking behavior likely related to the linguistic encoding of referents. That is, when the agent was mentioned explicitly and was thus more accessible, the authors observed a significant reduction of fixations to the depicted agent than when the agent was not mentioned or only indirectly referred to. These findings suggest that a discourse context can increase a referent’s accessibility and affect participants’ viewing behavior, similar to conceptual properties of a referent (such as animacy or imageability).

However, while derived accessibility effects have been well attested for the agent of an event, there is conflicting evidence regarding the accessibility of patients. On the one hand, Konopka and Meyer demonstrated that speakers’ structural choices were amenable to patient accessibility in a sentence production study (Konopka and Meyer, 2014). Speakers were more likely to produce passives (i.e., to start their utterances with the patient character) when the scene to be described was preceded by a priming picture that was semantically or associatively related to the patient. On the other hand, Ganushchak et al. (2017) did not observe any effect of patient accessibility, even when the patient was explicitly mentioned prior to a visually presented scene. Inconclusive results have also been obtained when derived patient accessibility was manipulated by means of visual preview (i.e., by presenting a picture of the patient prior to a scene, e.g., Prentice, 1967; Myachykov et al., 2012b, 2018). In one of the earliest studies conducted by Prentice (1967), participants were more likely to start their utterances with the patient when they had previously seen a cue slide depicting the patient character rather than the agent. While this study seems to reveal effects of patient accessibility, a number of caveats have to be taken into account. Most importantly, rather than eliciting descriptions of visual scenes, Prentice’s study was designed as a memory task. During a training phase, participants were repeatedly presented with a fixed combination of a cue slide (e.g., a man) and a target slide (e.g., a boy kicking a man), which they had to memorize. During test trials, participants saw a cue slide and then had to predict and describe the upcoming target slide without actually seeing it. One likely possibility—as the author acknowledges himself—is that participants responded strategically. Because no fillers were used, participants could always correctly guess and describe the upcoming scene by simply starting their utterances with the character depicted on the cue slide. It is thus not surprising that Prentice observed a remarkable effect of visual cueing on participants’ structural choices (i.e., the proportion of passives was more than 50% when the patient rather than the agent was depicted on the cue slide). Consequently, it remains questionable whether increasing patients’—rather than agents’—accessibility via preview can affect sentence production. The design of the current study involves varying patient accessibility and seeks to clarify this matter.

Two further studies examined effects of accessibility by means of visual preview (Myachykov et al., 2012b, 2018). In an attempt to disentangle whether the preview of a referent could affect participants’ structural choices beyond the mere allocation of attention to that referent, Myachykov et al. (2012b) tested participants in a sentence production experiment. Participants were asked to describe simple transitive events, e.g., a cowboy punching a boxer. Prior to the event scene, participants were either presented with the preview of one of the two referents (i.e., the agent or the patient) or with a meaningless attentional cue (i.e., a red dot that flashed up briefly in the same location where the primed character would appear). While participants were more likely to produce passives during attentional cueing of the patient, the visual preview of the patient did not additionally increase the rate of passives. Thus, Myachykov and colleagues did not observe any difference between the uninformative cue and the preview of a character, leading them to conclude that the accessibility of a referent is not decisive for participants’ structural choices (Myachykov et al., 2012b, 2018).

However, while Myachykov and colleagues carefully distinguished between effects of accessibility and visual attention, yet another factor seems to play an important role regarding visually elicited sentence production. In particular, there is recent evidence that the visuospatial position of a referent (i.e., whether a patient is depicted to the right or to the left of an agent) can affect speakers’ structural choices (Esaulova et al., 2019). Esaulova et al. (2019) examined the effect of position in a sentence production experiment using eye-tracking. German-speaking participants were asked to describe scenes depicting simple transitive events with two characters (e.g., a fisherman filming a clown). On half of the trials, the patient was located on the right of the agent, whereas on the other half the patient was located on the left of the agent. The results revealed that participants were faster in initiating their event descriptions when the patient appeared on the right, rather than on the left of the agent (Esaulova et al., 2019). Participants were also more inclined to produce passives when the patient character was located on the left than on the right, demonstrating that the position of referents exerts a substantial effect on speakers’ structural choices (for a recent replication in speakers of Russian, see Pokhoday et al., 2019).

Given these findings (i.e., similar effects of both accessibility and position on sentence structure), the visuospatial arrangement of characters seems directly relevant to the study of referent accessibility. Yet, so far, previous work on accessibility has not considered potential influences of position. Consequently, the effect of referent position on accessibility is currently unknown. The present study sought to fill this gap. In particular, our goal was to investigate the interplay between accessibility and character position by focusing on structural choices, speech onsets, and looking behavior as dependent measures. On the one hand, it is possible that the position of a referent is irrelevant to effects of accessibility. On the other hand, the accessibility of a referent might be affected by its position in a scene. This latter option could have important consequences for the study of accessibility. Because previous work on referent accessibility has merely counterbalanced the position of characters (Myachykov et al., 2012b, 2018), effects of position may have cancelled out effects of accessibility without being noticed. To elucidate this issue and to study the interplay between effects of referent position and accessibility, we combined manipulations of both factors within the same study. We manipulated patient accessibility by means of visual preview (i.e., derived accessibility). Additionally, we manipulated the animacy status of the patient. As summarized above, animate referents have repeatedly been shown to be more accessible than inanimate ones (e.g., Prat-Sala and Branigan, 2000; Van Nice and Dietrich, 2003; Tanaka et al., 2011), rendering animacy a prime factor influencing a referent’s inherent accessibility. Because effects of animacy have been well established even for patients (e.g., Prat-Sala and Branigan, 2000; Van Nice and Dietrich, 2003), we sought to compare these effects to our manipulation of derived accessibility (i.e., the visual preview of the patient character). To what degree are the effects of patient preview similar to—or different from—well-established effects exerted by animacy? Are both types of accessibility susceptible to potential influences of character position? To address these open questions, we studied effects of referent position along with manipulations of both derived and inherent accessibility.

We tested participants in an eye-tracking experiment where they had to describe scenes depicting simple events with an agent and a patient. To examine potential effects of derived patient accessibility, event scenes were either preceded by a referential cue (i.e., the visual preview of the patient character) or no cue. Crucially, unlike in previous studies (Myachykov et al., 2012b, 2018), the cue was presented centrally because we were interested in the specific contribution of referent accessibility without any confounding spatial information. Referent accessibility has previously been associated with facilitated subject selection and the resulting structural choice—a preference for subject-first passive rather than active structures (e.g., Bock et al., 1992; Prat-Sala and Branigan, 2000; Christianson and Ferreira, 2005). Therefore, if referential cueing served to increase the accessibility of the patient, we would expect participants to produce more passives in the cueing condition than in the no-cueing condition. It is also plausible to assume that more accessible referents would influence sentence planning times, so that we would expect shorter speech onset times for patient-first passive sentences after visually previewing patients compared to no previewing. We therefore considered participants’ speech onsets, providing more detailed insights into processes of utterance planning. In addition to language production, we analyzed the time course of speakers’ looking behavior to examine the potential effect of patient accessibility more closely (cf. Ganushchak et al., 2017). Similar to Ganushchak et al. (2017), we examined participants’ gaze patterns to the agent and patient character. In line with previous observations, the increase of a referent’s accessibility should lead to a reduction of fixations to that same referent because it is already active in the speaker’s mind (and does not have to be looked at again). Consequently, if speakers’ looking behavior were susceptible to the accessibility of the patient, we would expect to observe a reduction of fixations to the patient after cueing compared to the no-cueing condition (cf. Ganushchak et al., 2017 for accessibility effects of agents). To relate effects of derived accessibility (i.e., visual preview) to effects of inherent accessibility, we also manipulated the animacy status of the patient. In keeping with previous findings on referent animacy as an accessibility-related factor (Prat-Sala and Branigan, 2000; Van Nice and Dietrich, 2003), we expected participants to produce more passives when the patient was animate than when it was inanimate.

Beyond effects of patient accessibility, we examined whether sentence production was affected by the visuospatial arrangement of the depicted characters by manipulating the position of agents relative to patients (on the right in half of the experimental trials and on the left in the other half). Based on previous findings (e.g., Esaulova et al., 2019; Pokhoday et al., 2019), we expected participants to produce more passives after left-positioned than right-positioned patients. Unlike previous studies, we also directly related effects of position to the effects of accessibility. On the one hand, the accessibility of referents rendered via cueing and animacy could be unaffected by the position of referents. In this case, we should see no interaction between these factors. On the other hand, if accessibility-related factors were amenable to referent position, position should modulate effects of referential cueing and/or animacy, resulting in an interaction.

## Materials and Methods

### Participants

Forty-seven German native speakers (41 females, 6 males; mean age 22.21 years, SD = 3.12 years) were recruited from a database of volunteers at the University of Cologne, Germany, to participate in the experiment for either course credit or a monetary compensation. None of them reported any language or attention disorders, and all had normal or corrected-to-normal vision. All participants signed an informed consent form before starting the experiment. Ethical approval of the study was granted by the Ethics Commission of Cologne University’s Faculty of Medicine.

### Materials and Design

**FIGURE 1:**
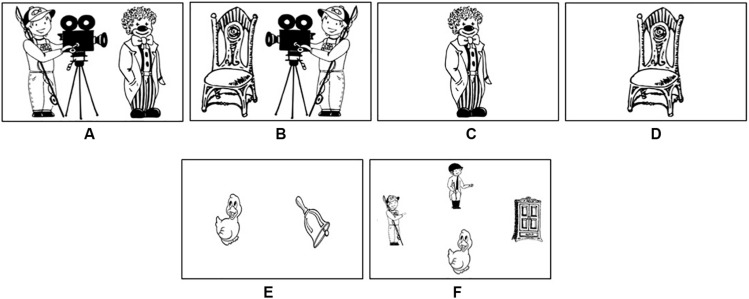
Examples of experimental **(A,B)** and filler items **(E)**, referential cues **(C,D)**, and a picture used in the familiarization task **(F)**.

A set of 56 black-and-white pictures of transitive event scenes was used as experimental items (Supplementary Table S1). Event scenes included an animate agent and an animate (28 items, Figure 1A) or an inanimate (28 items, Figure 1B) patient. All of the depicted characters were matched in size, the distance between agents and patients was kept constant, as well as contrasts, and the number of detail in these figures across items. Each event scene was represented by two mirror images, so that patients in the same event scene appeared to the right (28 items, Figure 1A) and to the left (28 items, Figure 1B) of agents. For each mirrored event scene, a picture of the patient in the center of the screen was created to serve as a referential cue (112 cueing items, Figures 1C,D) that was presented shortly before the corresponding event scenes in the cueing condition. A set of 56 pictures with depictions of two objects placed next to one another but with no event involved served as fillers (e.g., Figure 1E), half of which appeared following a picture of one of the objects in the center of the screen (Supplementary Table S2)^1^.

The experimental design included ANIMACY (animate/inanimate patient) as a within-subjects and between-items factor, as well as two within-subjects and within-items factors: POSITION (patient to the right/left of agent) and CUEING (cueing/no-cueing of patient). This resulted in eight conditions that were equally distributed among four lists with 56 items per list. Each participant saw one list and each item in one condition only. These lists were presented in seven blocks that consisted of eight experimental and eight filler items. Each of the blocks was preceded by a familiarization task, serving to ensure that participants could easily recognize the characters and objects that would later appear in the depicted scenes. The familiarization task consisted of responding to questions like “Where is (e.g., the duck)?” by pressing arrow keys while viewing pictures with four depictions of nouns that would later appear in the experimental and filler items of that block (four pictures per block, Figure 1F).

### Procedure

After participants signed a consent form, the Miles test was performed to determine participants’ eye dominance, and they were seated in front of an LCD monitor at a distance of 60 cm. Eye-movement data were recorded from the dominant eye using an EyeLink 1000 Plus eye tracker (SR Research Ltd.) at a sampling rate of 500 Hz. To ensure the accuracy of recordings, a nine-point calibration procedure was performed before the experiment began and repeated as needed during the experiment. A drift-correction screen with a target in the center of the screen appeared before each trial to verify if participants’ fixations on the target were accurate. At the beginning of the experimental session, participants were asked to wear a PC-headset Hama “Fire Starter” with a boom microphone that served to record speech data with a frequency range of 50 to 5,000 Hz.

**FIGURE 2:**
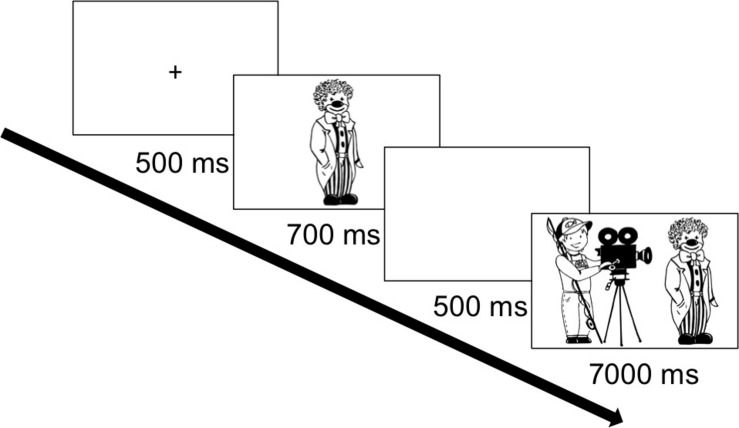
An illustration of the paradigm used in the experiment (cueing condition).

Participants were instructed via a stereo headphone to describe pictures of two entities depicted on the screen in one sentence. They were shown a picture of a transitive event and heard an example of an active voice sentence describing the scene [*Die Ärztin wiegt den Zauberer* (“The_NOM_ doctor weighs the_ACC_ magician”)], as well as an example of a passive voice sentence [*Der Zauberer wird von der Ärztin gewogen* (“The_NOM_ magician is weighed by the_DAT_ doctor”] and a description with a topicalized object [*Den Zauberer wiegt die Ärztin* (“The_ACC_ magician weighs the_NOM_ doctor”)]. All participants heard these three examples of syntactic structures that could be used to describe the scene. However, the presented scene included a feminine and a masculine referent, whereas only masculine characters (different from the one presented during the instruction) were included in the experimental items. Participants were also shown a picture displaying two objects next to one another (as in the filler items) and heard examples of descriptions using locative sentences [*Der Stern ist unter dem Mond* (“The star is below the moon”) and *Der Mond ist über dem Stern* (“The moon is above the star”)]. Then they were provided with a practice block that contained a familiarization task (as described above in *Materials and Design*) and two practice items to make sure participants understood the task. After this, the seven experimental blocks were presented. In the cueing condition, the presentation of experimental items is as shown in Figure 2: First, a fixation cross (500 ms) appeared in the center of the screen, and then a 700-ms referential cue was presented followed by a 500-ms blank screen to avoid an animation effect, and finally the event scene was presented and had to be described by participants. In the noncueing condition, the fixation cross was followed by a blank screen (1,200 ms), after which the event scene was presented. The entire experimental session lasted approximately 45 min.

### Data Analysis

Speech data were prepared for the statistical analyses using Praat software (Boersma and Weenink, 2017) to identify speech onset times and types of produced utterances. For the analysis of speech onset times, 0.7% of data were excluded because of recording issues (e.g., coughing or task-unrelated speech before the initiation of scene descriptions). Gaze data were collected for two rectangular interest areas of the same size that covered agent and patient drawings in scenes. The analyzed early measure of gaze patterns was the probability of first saccades to patients upon the scene onset. Later measures included the percentage of fixation times on patients and agents in two time windows. The first one covered the total fixation times from the scene onset until the average speech onset (1,673 ms). The second one excluded the initial fixations, starting from 400 ms after the onset of scenes and ending with the average speech onset. The later time window starting with 400 ms was selected for a better comparability with previous studies of eye-movement patterns during speech production using similar experimental tasks (Ganushchak et al., 2017).

The statistical analyses of obtained speech and gaze data included mixed-effects modeling in R (RStudio, 2017) using lme4 package (Bates et al., 2014) and lmerTest package to obtain *p* values for the observed effects (Kuznetsova et al., 2015). Continuous data (e.g., speech onset times) were analyzed with mixed-effects linear regression using lmer function. The appropriate data transformation was determined by means of the Box-Cox procedure (Box and Cox, 1964; Osborne, 2010) and resulted in a reciprocal square root transformation of active utterance onset data, a natural logarithmic transformation of passive utterance onset data, and a fourth root transformation of data reflecting the percentage of time spent on patients and agents 400 ms after scene onsets and prior to speech. Binomial data (e.g., probability of first saccades to patients) were analyzed with mixed-effects logistic regression using glmer function. The fixed effects in the models were ANIMACY, POSITION, CUEING, and the interactions between them. These categorical predictors were assigned sum-coded contrasts (Barr et al., 2013; Levy, 2014). Participants and items were included as random effects (Baayen et al., 2008). The best-fitting model was selected among converging models (built systematically reducing the maximal structure) based on the lowest AIC value. The follow-up contrast analyses for interactions were computed by refitting the models with respective factors as dummy-coded variables. In addition, Welch *t* test was computed to account for unequal sample sizes and variances when comparing speech onset times of passive and active sentences, as well as the gaze time spent on agents and patients before active and passive descriptions were produced.

## Results

### Speech

Active voice descriptions were produced in 82.4% of cases, with the remaining 17.6% being full passive voice utterances. The analysis of probability of passivizations (Table 1) revealed a main effect of POSITION, a main effect of CUEING, and an interaction between these factors. Scenes with left-positioned patients resulted in more produced passives (mean = 0.20) compared to scenes with right-positioned patients (mean = 0.16). Cueing patients also resulted in an increased probability of passive scene descriptions (mean = 0.25) compared to a no-cueing condition (mean = 0.11). These main effects were further qualified by an interaction between POSITION and CUEING. The follow-up contrast analyses showed that the probability of produced passive utterances was significantly higher after cueing (mean = 0.26) than no-cueing (mean = 0.14) when patients appeared to the left of agents, *b* = 1.68, SE = 0.60, *z* = 2.80, *p* = 0.005. At the same time, the difference between cueing (mean = 0.23) and no-cueing (mean = 0.08) conditions did not reach significance for scenes with right-positioned patients, *b* = 1.29, SE = 0.84, *z* = 1.54, *p* = 0.124^2^. Probabilities of produced passives also did not differ between right- and left-positioned patients in both cueing (mean_right_ = 0.23, mean_left_ = 0.26; *b* = −0.60, SE = 0.48, *z* = −1.24, *p* = 0.215) and no-cueing (mean_right_ = 0.08, mean_left_ = 0.14; *b* = −0.53, SE = 0.78, *z* = 0.68, *p* = 0.498) conditions.

**TABLE 1:** Main effects and interactions from the mixed-effects logistic regression model on the probability of passive utterance production (model < - glmer [DV ∼ animacy × position × cueing + (1 | participant) + (1 | item)]).

	*b*	SE	*z*	*p*
Intercept (estimated grand mean)	–3.54479	0.51196	–6.92	< 0.001**
Animacy (animate–inanimate)	0.14335	0.07925	1.81	0.071
Position (left–right)	0.30218	0.07280	4.15	< 0.001**
Cueing (noncued–cued)	–0.88203	0.07831	–11.26	< 0.001**
Animacy × position	–0.01575	0.07458	–0.21	0.833
Animacy × cueing	–0.05123	0.07921	–0.65	0.518
Position × cueing	0.16208	0.07239	2.24	0.025*
Animacy × position × cueing	–0.11817	0.07385	–1.60	0.110

*p < 0.05; **p < 0.001.

Active utterances were on average initiated later (mean = 1,667.13, SD = 594.56, SE = 12.79) than passive ones (mean = 1,567.88, SD = 693.16, SE = 32.53), *t*(600.86) = −2.84, *p* = 0.005^3^. The analysis of onsets of active utterances upon the presentation of event scenes (Table 2) resulted in main effects of CUEING, POSITION, and an interaction between ANIMACY and CUEING. The initiation of active utterances was delayed in the noncueing (mean = 1,727.59, SD = 628.62, SE = 18.35) compared to the cueing condition (mean = 1,595.34, SD = 543.04, SE = 17.28), as well as when patients were to the left (mean = 1,700.39, SD = 615.32, SE = 18.96) rather than to the right (mean = 1,635.52, SD = 572.63, SE = 17.20) of agents. The main effect of CUEING was qualified by a significant interaction between CUEING and ANIMACY. Follow-up analyses showed a significant reduction in speech onset times of active descriptions for scenes with animate patients when those were cued (mean = 1,572.82, SD = 525.03, SE = 23.89) rather than noncued (mean = 1,742.53, SD = 597.02, SE = 24.73), *b* = 0.001, SE = 0.0003, *t* = 3.80, *p* < 0.001. No other contrasts within the interaction revealed significant differences.

**TABLE 2:** Main effects and interactions from the mixed-effects linear regression model on the speech onset times of active utterances (model < - lmer[1/sqrt(DV) ∼ animacy × position × cueing + (1 + animacy + position | participant) + (1 + animacy | item)]).

	*b*	SE	*t*	*p*
Intercept (estimated grand mean)	0.02530	0.00036	70.92	< 0.001***
Animacy (animate–inanimate)	–0.00012	0.00009	–1.38	0.174
Position (left–right)	–0.00025	0.00007	–3.49	0.001**
Cueing (noncued–cued)	–0.00047	0.00006	–7.38	< 0.001***
Animacy × position	0.00000	0.00007	0.01	0.989
Animacy × cueing	–0.00017	0.00008	–2.22	0.027*
Position × cueing	–0.00006	0.00006	–1.03	0.302
Animacy × position × cueing	0.00008	0.00006	1.20	0.229

*p < 0.05; **p < 0.01; ***p < 0.001.

The analysis of onsets of passive utterances (Table 3) showed a main effect of CUEING. The initiation of passive utterances was significantly faster when patients were cued (mean = 1,435.85, SD = 671.08, SE = 37.81) compared to when they were not (mean = 1,867.08, SD = 650.01, SE = 55.13).

**TABLE 3:** Main effects and interactions from the mixed-effects linear regression model on the speech onset times of passive utterances (model < - lmer[log(DV) ∼ animacy × position × cueing + (1 + animacy + cueing | participant) + (1 | item)]).

	*b*	SE	*t*	*p*
Intercept (estimated grand mean)	7.37968	0.04463	165.36	< 0.001*
Animacy (animate–inanimate)	–0.00108	0.01898	–0.06	0.955
Position (left–right)	–0.02775	0.01569	–1.77	0.078
Cueing (noncued–cued)	0.12109	0.02696	4.49	< 0.001*
Animacy × position	0.01527	0.01611	0.95	0.344
Animacy × cueing	–0.00972	0.01710	–0.57	0.570
Position × cueing	–0.00928	0.01587	–0.59	0.559
Animacy × position × cueing	–0.01834	0.01626	–1.13	0.260

*p < 0.001.

### Gaze

#### General Overview of Gaze–Speech Correspondence

**FIGURE 3:**
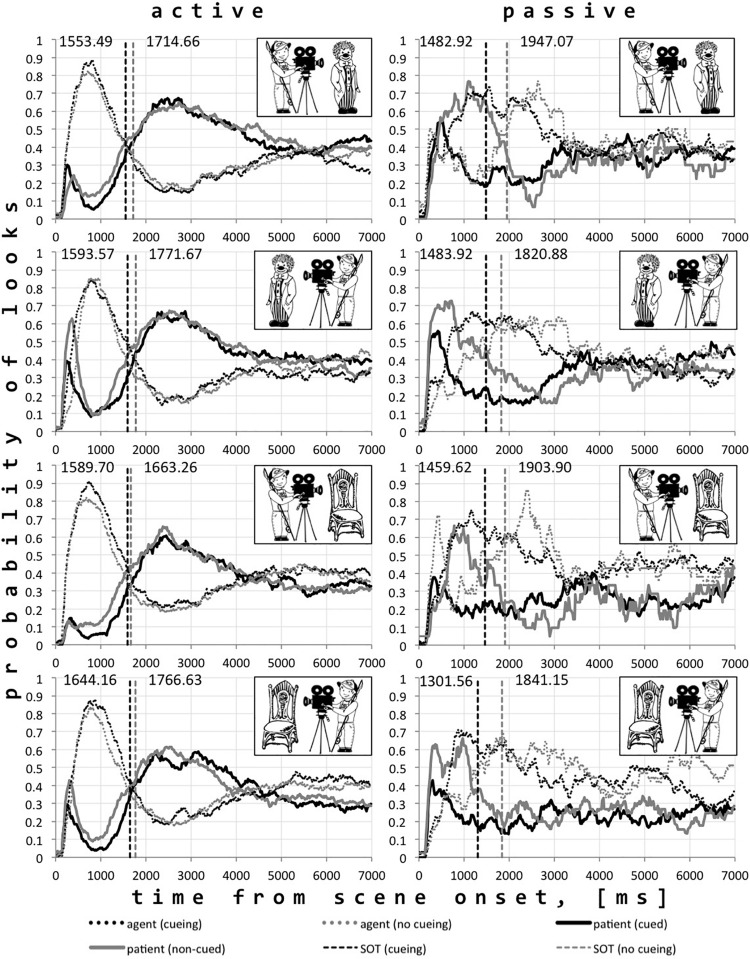
The probability of looks to agents and patients corresponding to active and passive utterances and average speech onsets in each condition.

The analysis of the time window from the scene onset until average speech onset showed that the percentage of gaze time spent on agents was significantly higher than that spent on patients (Table 4) when produced passive and active utterances are considered separately, *t*_passive_ (923.96) = 7.23, *p* < 0.001; *t*_active_ (4,226.1) = 75.30, *p* < 0.001. Nevertheless, more gaze time was spent on patients when passive rather than active descriptions were produced, *t*_patients_ (565.79) = −13.25, *p* < 0.001. At the same time, more gaze time was spent on agents when active rather than passive descriptions were produced, *t*_agents_ (607.16) = 13.06, *p* < 0.001. Figure 3 represents the time course of looks to patients and agents in all conditions when active and passive utterances were produced.

**TABLE 4:** Means (*SD* and *SE*) of the percentage of gaze time spent on agents and patients from scene to speech onsets when active and passive descriptions are produced.

	Active	Passive
Agents	0.57 (0.18, 0.003)	0.43 (0.22, 0.01)
Patients	0.18 (0.16, 0.003)	0.32 (0.22, 0.01)

#### Looking Patterns Due to Experimental Factors

The analysis of first saccades to patients upon the onset of the event scene (Table 5) revealed main effects of ANIMACY and POSITION, as well as an interaction between POSITION and CUEING. The probability of first saccades to patients was higher for animate (mean = 0.36) than inanimate (mean = 0.28) and for left- (mean = 0.43) than right-positioned (mean = 0.21) patients. The follow-up contrast analyses of the POSITION × CUEING interaction showed a significantly higher probability of first saccades to both cued and noncued patients when they were left- (mean_cued_ = 0.37; mean_noncued_ = 0.50) rather than right-positioned (mean_cued_ = 0.25; mean_noncued_ = 0.18), *b*_cued_ = −0.69, SE = 0.23, *z* = −2.96, *p* = 0.003; *b*_noncued_ = −1.76, SE = 0.26, *z* = −6.79, *p* < 0.001. Moreover, left-positioned patients received significantly fewer first saccades after cueing than no cueing, *b* = −0.58, SE = 0.19, *z* = −3.05, *p* = 0.002. The opposite was true for right-positioned patients, which received more first saccades after cueing than no cueing, *b* = 0.51, SE = 0.19, *z* = 2.73, *p* = 0.006.

**TABLE 5:** Main effects and interactions from the mixed-effects logistic regression model on the probability of first saccades to patients (model < - glmer[DV ∼ animacy × position × cueing + (1 + animacy + position | participant) + (1 | item)]).

	*b*	SE	*z*	*p*
Intercept (estimated grand mean)	–0.92533	0.08867	–10.44	< 0.001*
Animacy (animate–inanimate)	0.23342	0.05884	3.97	< 0.001*
Position (left–right)	0.61058	0.10177	6.00	< 0.001*
Cueing (noncued–cued)	0.02148	0.04722	0.46	0.649
Animacy × position	–0.00274	0.05047	–0.05	0.957
Animacy × cueing	–0.05333	0.05327	–1.00	0.317
Position × cueing	0.27533	0.04730	5.82	< 0.001*
Animacy × position × cueing	0.02990	0.04899	0.61	0.542

*p < 0.001.

The analysis of the percentage of fixation time spent on patients in the later time window—from 400 ms after the onset of scenes until average speech onset (Table 6)—resulted in main effects of POSITION, CUEING, and ANIMACY. The mean patterns were similar to those observed for first fixations: more gaze time was spent on left-positioned (mean = 0.22, SD = 0.22, SE = 0.01) than right-positioned (mean = 0.21, SD = 0.22, SE = 0.01), as well as noncued (mean = 0.26, SD = 0.23, SE = 0.01) than cued (mean = 0.18, SD = 0.21, SE = 0.01) patients. There were also longer fixations on animate (mean = 0.24, SD = 0.23, SE = 0.01) than inanimate (mean = 0.19, SD = 0.21, SE = 0.01) patients.

**TABLE 6:** Main effects and interactions from the mixed-effects linear regression model on the percentage of fixation times on patients 400 ms after the onset of scenes and before speech onset (model < - lmer[(DV) ^0.25 ∼ animacy × position × cueing + (1 + animacy + cueing | participant) + (1 | item)]).

	*b*	SE	*t*	*p*
Intercept (estimated grand mean)	0.50080	0.01873	26.74	< 0.001*
Animacy (animate–inanimate)	0.03830	0.00732	5.23	< 0.001*
Position (left–right)	0.02106	0.00595	3.54	< 0.001*
Cueing (noncued–cued)	0.06405	0.00902	7.10	< 0.001*
Animacy × position	–0.00067	0.00625	–0.11	0.914
Animacy × cueing	–0.00910	0.00699	–1.30	0.193
Position × cueing	0.00720	0.00595	1.21	0.226
Animacy × position × cueing	–0.00249	0.00625	–0.40	0.690

*p < 0.001.

## Discussion

The central goal of our study was to examine effects of derived and inherent patient accessibility in descriptions of event scenes. Our results show that visually cueing the patient affected participants’ structural choices, speech onset times, and gaze patterns. Importantly, we found that effects of cueing were not independent of the visuospatial position of the patient as evidenced by significant interaction effects. These findings support the notion that character position can modulate other accessibility-related factors (i.e., visual preview). At the same time, we observed differences between the different accessibility-related factors: Effects of position and animacy affected participants’ gaze patterns in different ways than did cueing. These findings suggest that different accessibility factors may involve different underlying processes of sentence planning. Finally, we provide evidence that derived and inherent accessibility factors interact, affecting sentence production to a different extent. In the following, we will discuss our findings as well as their implications in more detail.

### Effects of Derived Accessibility Factors

#### Referent Cueing

Previous studies have yielded mixed results with respect to patient accessibility (e.g., Myachykov et al., 2012b, 2018; Konopka and Meyer, 2014; Ganushchak et al., 2017). For instance, whereas Konopka and Meyer (2014) demonstrated that speakers were more likely to produce passives when scene descriptions were preceded by a priming picture related to the patient, Ganushchak et al. (2017) did not observe any effect of patient accessibility—neither on participants’ structural choices nor on their gaze patterns. Yet, our results show that cueing patients affected both sentence production and looking behavior. Unlike Ganushchak et al. (2017), who merely observed effects of agent but not patient accessibility on gaze patterns, we found participants’ looking behavior to be amenable to effects of patient accessibility. Participants in our study were less likely to fixate the patient character after cueing than when no preactivation occurred in the later time window (400–1,673 ms). In the earliest time window (i.e., first saccades), cueing also modulated participants’ looking patterns, resulting in fewer fixations on patient referents when they were positioned on the left. This is in contrast with findings by Ganushchak et al. (2017), who observed effects of agent accessibility only in the later time window (400–1,400 ms) but no accessibility effects earlier (0–400 ms)^4^. Ganushchak and colleagues therefore argue that accessibility has an effect on the linguistic encoding of referents rather than on sentence planning. However, our findings suggest that effects of patient accessibility may not be limited to the linguistic encoding of the patient character but that these effects also pertain to sentence planning. While our results and those of Ganushchak and colleagues seem in opposition, it should be stressed that a variety of factors were different in the two studies, rendering a direct comparison difficult. For instance, unlike Ganushchak and colleagues, who manipulated accessibility in terms of the preceding discourse, we made use of visual cueing. Depending on the condition, discourse context used by the authors in their study activated either conceptual, lexical, and phonological information (literal mentioning of the event character in a story prior to scene) or conceptual information (a story associatively related to the event character). Both conditions thus tapped into mental representations of characters and did so via linguistic means. The referential cueing in our study also aimed at activating conceptual information but did so via visual means. The processes involved in the visual inspection of the referent prior to the activation of its mental representation were thus qualitatively different from those in Ganushchak et al. (2017). Not only may the activation of a mental representation based on linguistic input be different from that based on visual input, but also the very eye-movement patterns upon the presentation of a scene may differ after a heard story compared to a previewed referent. Furthermore, we kept the size of the depicted agent and patient characters comparable, whereas the two characters in Ganushchak’s study often differed in size (e.g., a bigger frog swallowing a much smaller fly, cf. Ganushchak et al., 2017). In summary, our results demonstrate that previewing patients seemed to be effective in making them more accessible than when no cueing was provided. This finding was further corroborated by effects of patient accessibility on sentence production. Participants were faster to initiate utterances when they had previously encountered the patient character by means of visual preview. They were also more likely to start their utterances with the patient (i.e., produce passives) when the patient character had been cued.

Our findings contrast with those by Myachykov et al. (2012b) and Myachykov et al. (2018), who argue against effects of accessibility on structural choices. It should be stressed that—like in our study—Myachykov and colleagues found an increase of English-speakers’ passivizations when the patient character had been cued as opposed to a noncueing condition. However, unlike in our study, the goal of Myachykov and colleagues was to assess the specific contributions of accessibility (i.e., referential priming) beyond the mere allocation of attention (i.e., priming by means of a non-symbolic cue). Because this contrastive approach revealed no difference between the proportion of passives during referential and attentional priming, the authors concluded that structural choices are not influenced by effects of referent accessibility. Although the focus of our study was not on a comparison between referential and attentional manipulations, our results nevertheless challenge some of the conclusions drawn by Myachykov and colleagues: Regarding participants’ eye movements (i.e., an indicator of visual attention), we found that the preview of the patient led to a reduction of fixations to the patient character in the subsequently presented scene. Yet, although participants’ visual attention was to a lesser extent allocated to the patient, participants still produced more passives when the patient had been cued. These results suggest that patient accessibility exerted an effect beyond attentional processes (i.e., decreased attention but more passives), contrasting with findings by Myachykov and colleagues. What could cause these divergent observations? It is possible that cross-linguistic differences play a role. In particular, attentional cueing seems to exert a comparatively strong effect in speakers of English, as reflected by the high rate of passives produced in this condition (around 20–30% of passives, see e.g., Myachykov et al., 2018, also see Gleitman et al., 2007; Myachykov et al., 2011, 2012a). By contrast, attentional cueing does not seem to be equally effective in languages that make use of a richer set of inflectional morphology (i.e., case markings on nouns, Esaulova et al., 2019 for German, also see Hwang and Kaiser, 2015 for Korean). For instance, unlike speakers of English, German speakers did not produce significantly more passives when their attention was cued by a visual stimulus, despite the fact that cueing influenced German speakers’ looking behavior (Esaulova et al., 2019). When the attentional cue was presented for a longer duration, German speakers’ rate of passives increased to around 11% but still did not reach the high proportion of passives produced by English-speaking participants (Esaulova, Dolscheid, and Penke, n.d.). Differences in the effectiveness of attentional cueing (i.e., high rates of passives for English but not German speakers) may thus have mitigated potential effects of accessibility in speakers of English. In line with this explanation, a direct comparison of our results and those of Esaulova and colleagues (n.d.) reveals that German-speaking participants produced more passives after referential cueing (18%) than after attentional cueing (11%) in an otherwise equivalent design. These findings show that German speakers displayed effects of patient accessibility beyond the mere allocation of attention, contrasting with patterns observed in English-speaking participants. Whereas future studies should examine cross-linguistic variation more directly, our results suggest that differences between English and German might influence the assessment of accessibility effects.

### Referent Position

In addition to cueing as a derived accessibility factor, another central factor considered in our study was the position of patients in scenes. A number of previous studies have demonstrated that the position of agents and patients plays an important role for participants’ scene encodings (Dobel et al., 2007b) and that it affects the way speakers represent events (e.g., Chatterjee et al., 1999). However, only recently, there is evidence that this factor is also critical for speakers’ sentence production (Esaulova et al., 2019; Pokhoday et al., 2019). For instance, Esaulova et al. (2019) showed that speakers of German were more likely to produce passives when the patient character was located on the left rather than on the right of the agent (for further discussion and the possible role of reading/writing direction, see e.g., Esaulova et al., 2019; Dobel et al., 2007a). In the present study, we replicated these findings by showing that the visuospatial arrangement of event characters affected both participants’ eye movements and sentence production. Regarding eye gaze, participants spent more time fixating the patient character when it was located on the left than on the right. This was reflected in both initial saccades to patients, as well as later fixations on them. In keeping with previous findings (e.g., Esaulova et al., 2019), German-speaking participants seemed to expect the leftmost position of a scene to be taken up by the agent rather than by the patient character. This left-to-right preference was also reflected in participants’ sentence production. Participants were slower to initiate their responses when the patient was located on the left, rather than on the right. Furthermore, participants were more likely to start their utterances with the patient when it was depicted on the left than on the right (see Esaulova et al., 2019; Pokhoday et al., 2019 for converging evidence). These findings suggest that participants tended to align the leftmost position with the subject function, leading to a greater proportion of passives when patients were depicted on the left.

Crucially, given its influence on participants’ structural choices, our results suggest that referent position can also be conceived of as an accessibility-related factor, influencing participants’ sentence production in similar ways as the preview of a referent. It is interesting to note, however, that referent cueing and position manipulations resulted in opposite gaze patterns. While previewing patients led to fewer looks on them than when no previewing occurred, placing patients on the left rather than on the right led to more looks. This difference suggests that making a distinction between the mechanisms underlying each of the two accessibility-related factors may be meaningful. The previewing of a referent may activate the corresponding mental representation so that no further visual inspection is necessary once the scene with that referent appears on the screen. In contrast, the effect of referent position may be better explained by highly automatized looking habits, where the scene tends to be inspected from left to right by speakers of a language with a left-to-right writing system. However, even though this seems a plausible interpretation of the nature of the observed difference in gaze responses to the two factors, it has to be regarded with caution and should for now remain a speculation.

Beyond comparing effects of visual preview and position one central goal of the present study was to examine the interplay between the two factors in more detail. Are effects of referential preview and position independent from each other or do they interact? In other words: Is the accessibility-related factor “preview” susceptible to the positioning of the patient character? Our results answer in the affirmative. In particular, we observed a significant interaction between the location of the patient character and its preview as evidenced by both sentence production and viewing behavior. While overall left-positioned patients attracted more looks than right-positioned patients, referential cueing exerted different effects depending on the position of the patient character. Thus, only for left-positioned patients, cueing as opposed to noncueing led to a significant decrease of first fixations to the patient character. This finding suggests that the accessibility-related factor cueing was effective only when the patient was located on the left. For right-positioned patients, on the other hand, the reverse held true. More first saccades were spent on right-positioned patients when they were cued rather than not. A similar interaction was observed for participants’ structural choices. Participants produced more passives after cueing than noncueing for left-positioned but not for right-positioned patients, demonstrating that effects of cueing were restricted to left-positioned patients. Taken together, our findings show that accessibility effects due to cueing are not independent of the location of a referent and that changes in participants’ structural choices and eye gaze induced by cueing were particularly pronounced for left-positioned patients. These findings support the idea that the position of a character can modulate a referent’s accessibility status, emphasizing the importance of the factor position for referent accessibility.

The observed interaction between position and cueing also challenges conclusions about sentence planning strategies. Broadly speaking, two different types of incremental planning have been proposed in the field: linear (or lexical) versus hierarchical (or structural) incrementality (e.g., Bock et al., 2004). Whereas linear incrementality predicts that a speaker plans the preverbal message one concept at a time (e.g., Gleitman et al., 2007), hierarchical incrementality assumes that sentence formulation begins with the generation of a broader sentence plan that already captures the relationship between the various event characters (Bock et al., 2004). Crucially, the two theories make different predictions regarding effects of patient accessibility during the production of active sentences in an early time window (i.e., before 400 ms, cf. Ganushchak et al., 2017). Linear incrementality predicts no effects of patient accessibility because the patient is only planned in a separate (i.e., later) increment after the agent. By contrast, hierarchical incrementality predicts effects of patient accessibility because speakers already plan a larger preverbal message including information not only about the agent but also the patient. As outlined above, we observed effects of patient accessibility (i.e., referential cueing) even in an early time window (i.e., before 400 ms). While this could be taken as evidence in favor of hierarchical incrementality (i.e., speakers have extracted sufficient information about both referents including the later mentioned patient), this effect was only observed for left-located but not for right-located patients. This suggests that any conclusions about sentence planning strategies based on accessibility effects should be treated with caution because we show that cueing itself is susceptible to influences of position.

Beyond theoretical considerations, the interaction between position and cueing has important methodological consequences for the study of accessibility effects. Specifically, differences in the arrangement of event characters could be one of the driving forces underlying the heterogeneous findings reported for patient accessibility (e.g., Myachykov et al., 2012b, 2018; Ganushchak et al., 2017; vs. Konopka and Meyer, 2014). Because previous work has not taken effects of position into account (or merely counterbalanced the locations of characters, see e.g., Myachykov et al., 2012b), this factor may have obscured effects of accessibility without being recognized. In particular, counterbalancing the position of characters could have canceled out effects of accessibility because position can affect other accessibility-related factors (i.e., cueing).

Summarizing, our findings highlight the importance of controlling for effects of position, especially when testing other accessibility-related factors such as cueing. Furthermore, the position effect might also be relevant for experimental designs where referential cues are presented on the left or on the right but not in the center of the screen (see e.g., Myachykov et al., 2012b, 2018). While future studies should investigate whether the location of a referential cue can introduce similar biases as the position of a character in a scene, our findings indicate that it may be important to disentangle the location and the conceptual properties of cueing. Experiments that make use of referential cueing should thus examine accessibility effects without any confounding spatial information.

### Effects of Inherent Accessibility

Previous research has shown that animacy is a factor inherent to a referent and that it exerts a pervasive influence on referent accessibility (e.g., Prat-Sala and Branigan, 2000; Van Nice and Dietrich, 2003). Including this factor in our study allowed us to test whether animate patients were more accessible than inanimate ones. However, this hypothesis was only partly confirmed when speakers’ structural choices are regarded. Because the *p* value of this effect was 0.071, the effect was only significant under a directed hypothesis. More convincing evidence in favor of animacy was revealed by participants’ looking behavior where animate rather than inanimate patients were more likely to attract participants’ eye gaze. Taken together, these findings suggest that the animacy status of a patient exerted an influence on both eye gaze and sentence production, although the effect was smaller than anticipated. How can this finding be reconciled with the vast number of studies highlighting the importance of animacy for sentence production (e.g., Bock and Warren, 1985; Prat-Sala and Branigan, 2000; Branigan et al., 2008; Tanaka et al., 2011)? It should be stressed that instead of manipulating both agent and patient animacy, we exclusively manipulated patient characteristics. At the same time, other studies elicited passivizations most reliably in constellations with an inanimate agent (e.g., a man being hit by a swing, see e.g., Prat-Sala and Branigan, 2000). By contrast, the agent character in our study was always animate. Because the most contrastive combination—an animate patient with an inanimate agent—was not part of our design, this may have caused the attenuated effect observed in our sample.

### Comparison and Interplay Between Derived and Inherent Accessibility Factors

When comparing effects of visual preview to effects of animacy on participants’ gaze patterns, participants were less likely to fixate the cued patient character, but the opposite was true for animate patients (i.e., animates were fixated more during both the earliest and later time window). These findings point to differences regarding effects of the two accessibility-related factors. Because animacy has been shown to be of high importance for human beings even from infancy on (e.g., Poulin-Dubois et al., 1996), it also appears to capture participants’ attention during sentence planning, resulting in an increase of fixations. The effect of visual preview, by contrast, seems to involve a decrease of (visual) attention. While the application of the rather broad term “accessibility” implies that both cueing and animacy may work in a similar fashion, our findings suggest this conclusion is not viable. As our results show, the examined factors modulated accessibility in different ways, supporting the idea that accessibility should not simply be viewed as a monolithic property of a referent. Rather it has to be conceptualized as more graded and dynamic in nature. In line with this, we also found a significant interaction between animacy and referential cueing on participants’ speech onsets. Speakers produced active descriptions faster when animate patients were cued than when they were not. These results suggest that both accessibility-related factors—animacy and referential cueing—seem to affect participants’ sentence production. Crucially, however, the contribution of each of the two appears to be different, as indicated by the significant interaction. In this context, it is interesting to note that inherent accessibility did not appear to be susceptible to influences of position. That is, only cueing but not the animacy status of a patient interacted with the visuospatial arrangement of characters in a scene. It is thus possible that inherent properties of referents (like animacy) are more robust and less likely affected by transient information such as spatial position. However, this interpretation has to be treated with some caution because the absence of evidence (in this case the absence of an interaction between animacy and position) should not be taken as evidence of absence. Still, our findings demonstrate that cueing was amenable to effects of position, whereas the same did not apply to animacy. While future studies should investigate potential differences between the various facets of accessibility in more detail, our study provides the first evidence that referential cueing can be modulated by the location of event characters.

## Conclusion

The present study examined patient cueing, position, and animacy as accessibility-related factors that influence speakers’ sentence production and looking behavior when they describe visual event scenes. While both animacy status and referential cueing influenced participants’ sentence production, effects appeared to be more pronounced for the latter. In addition, we could show that the position of characters exerted a substantial effect on participants’ sentence production, thereby replicating recent findings (e.g., Esaulova et al., 2019; Pokhoday et al., 2019). Most critically, we found a significant interaction between position and referential cueing, demonstrating that the position of a referent can influence another accessibility-related factor (i.e., cueing). Our findings therefore emphasize the importance of controlling for character position in future sentence production experiments.

## Data Availability Statement

The datasets generated for this study are available on request to the corresponding author.

## Ethics Statement

The studies involving human participants were reviewed and approved by Ethics Commission of Cologne University’s Faculty of Medicine, University of Cologne. The patients/participants provided their written informed consent to participate in this study.

## Author Contributions

YE designed the study, collected and analyzed the data, and wrote the manuscript. MP and SD designed the study and wrote the manuscript. All authors contributed to the article and approved the submitted version.

## Conflict of Interest

The authors declare that the research was conducted in the absence of any commercial or financial relationships that could be construed as a potential conflict of interest.

## Footnotes

1 The pictures of objects were taken from websites that provide open source access to pictures and modified to create filler items (e.g., https://clker.com, https://pixabay.com, https://clipartpanda.com).

2 This might be due to the fact that there were overall fewer passive sentences produced for right-patient conditions (number of passives: 204) compared to left-patient ones (259).

3 This finding is not discussed in the present article, as it is beyond its scope. However, this pattern was observed in a series of experiments with a similar design and is covered in a separate paper (Esaulova, Dolscheid, and Penke, n.d.).

4 Note that while time windows in our study are not identical to those analyzed in Ganushchak et al. (2017), these differences are unlikely to be critical for a comparison of the results, because the comparison is made based on equivalent measures reflecting relatively early (before 400 ms) and later (after 400 ms) processes. Unlike Ganushchak and colleagues, who analyzed a time window of 0 to 400 ms, we analyzed participants’ first saccades after scene onset. Considering that a saccade takes between 100 and 350 ms to initiate (Carpenter, 1988; Gupta et al., 2019), our analysis of first saccades in fact corresponds to a time window of the same order as in Ganushchak, Konopka, and Chen 2017.
