# Supplementary material for: Referent Cueing, Position, and Animacy as Accessibility Factors in Visually Situated Sentence Production
Source: Front Psychol. 2020 Aug 27;11:2111. doi: 10.3389/fpsyg.2020.02111 (PMC7493676; doi:10.3389/fpsyg.2020.02111)
Supplement: Supplementary file 1 [file Table_1.DOCX]

Supplementary Material

**Supplementary Table 1.** The list of experimental items used as pictures.

| **Item** | **Agent** | **Action** | **Patient** |
| --- | --- | --- | --- |
| 1 | angler | to film | clown |
| 2 |  |  | chair |
| 3 | baker | to fish | cowboy |
| 4 |  |  | globe |
| 5 | outlaw | to weigh | anchor |
| 6 |  |  | monk |
| 7 | boxer | to push | thief |
| 8 |  |  | cupboard |
| 9 | butler | to film | anchor |
| 10 |  |  | farmer |
| 11 | fisher | to weigh | farmer |
| 12 |  |  | chair |
| 13 | forester | to fish | hammer |
| 14 |  |  | sheriff |
| 15 | gardener | to push | clown |
| 16 |  |  | sack |
| 17 | prisoner | to weigh | cowboy |
| 18 |  |  | suitcase |
| 19 | hunter | to push | angel |
| 20 |  |  | armchair |
| 21 | waiter | to water | basket |
| 22 |  |  | jester |
| 23 | king | to measure | bow |
| 24 |  |  | devil |
| 25 | runner | to water | stool |
| 26 |  |  | cook |
| 27 | painter | to measure | jester |
| 28 |  |  | cupboard |
| 29 | butcher | to film | stove |
| 30 |  |  | monk |
| 31 | pope | to water | angel |
| 32 |  |  | armchair |
| 33 | pastor | to weigh | hammer |
| 34 |  |  | knight |
| 35 | prince | to hit | saddle |
| 36 |  |  | devil |
| 37 | robber | to fish | stove |
| 38 |  |  | dwarf |
| 39 | reider | to hit | thief |
| 40 |  |  | basket |
| 41 | judge | to hit | globe |
| 42 |  |  | sheriff |
| 43 | shepherd | to weigh | stool |
| 44 |  |  | vampire |
| 45 | tailor | to fish | pirate |
| 46 |  |  | saddle |
| 47 | pupil | to hit | coffin |
| 48 |  |  | vampire |
| 49 | swimmer | to film | cook |
| 50 |  |  | coffin |
| 51 | sportsman | to film | suitcase |
| 52 |  |  | pirate |
| 53 | diver | to hit | sack |
| 54 |  |  | dwarf |
| 55 | tourist | to push | bow |
| 56 |  |  | knight |

**Supplementary Table 2.** The list of filler items used as pictures.

| **Item** | **Object 1** | **Position of object 1** | **Object 2** | **Position of object 2** |
| --- | --- | --- | --- | --- |
| 1 | ant | left | cup | right |
| 2 |  | top | frog | bottom |
| 3 | traffic light | left | kettle | right |
| 4 |  | top | owl | bottom |
| 5 | apple | left | fish | right |
| 6 |  | top | glasses | bottom |
| 7 | bear | left | duck | right |
| 8 |  | top | table | bottom |
| 9 | tree | left | frog | right |
| 10 |  | top | cup | bottom |
| 11 | glasses | left | apple | right |
| 12 |  | top | cat | bottom |
| 13 | elephant | left | giraffe | right |
| 14 |  | top | hat | bottom |
| 15 | duck | left | bell | right |
| 16 |  | top | bear | bottom |
| 17 | owl | left | traffic light | right |
| 18 |  | top | tiger | bottom |
| 19 | fish | left | cat | right |
| 20 |  | top | apple | bottom |
| 21 | fly | left | lamp | right |
| 22 |  | top | bird | bottom |
| 23 | frog | left | ant | right |
| 24 |  | top | tree | bottom |
| 25 | giraffe | left | mill | right |
| 26 |  | top | elephant | bottom |
| 27 | guitar | left | moon | right |
| 28 |  | top | mouse | bottom |
| 29 | bell | left | table | right |
| 30 |  | top | duck | bottom |
| 31 | hat | left | elephant | right |
| 32 |  | top | mill | bottom |
| 33 | cat | left | glasses | right |
| 34 |  | top | fish | bottom |
| 35 | kettle | left | tiger | right |
| 36 |  | top | traffic light | bottom |
| 37 | lamp | left | envelope | right |
| 38 |  | top | fly | bottom |
| 39 | mouse | left | guitar | right |
| 40 |  | top | penguin | bottom |
| 41 | moon | left | penguin | right |
| 42 |  | top | guitar | bottom |
| 43 | penguin | left | mouse | right |
| 44 |  | top | moon | bottom |
| 45 | cup | left | tree | right |
| 46 |  | top | ant | bottom |
| 47 | tiger | left | owl | right |
| 48 |  | top | kettle | bottom |
| 49 | table | left | bear | right |
| 50 |  | top | bell | bottom |
| 51 | envelope | left | lamp | right |
| 52 |  | top | bird | bottom |
| 53 | bird | left | fly | right |
| 54 |  | top | envelope | bottom |
| 55 | mill | left | hat | right |
| 56 |  | top | giraffe | bottom |
